# Supplementary material for: Modeling and implementation of a real-time digital twin for the Stewart platform with real-time trajectory computation
Source: PeerJ Comput Sci. 2025 May 20;11:e2892. doi: 10.7717/peerj-cs.2892 (PMC12192767; doi:10.7717/peerj-cs.2892)
Supplement: Supplemental Information 1 [file peerj-cs-11-2892-s001.zip › StewartPro Shadow/intro.docx]

The main part of the thesis covered is still at the Through-Life Engineering Services Centre, Cranfield University, for the purpose of carrying out the next relevant research, including the physical entities of the Stewart platform shown in the photographs, the Stewart Digital Twin Connection communication protocols, the data interaction code, etc., which, due to university as well as laboratory regulations, the Due to university and lab regulations, all of the above materials are stored in the lab's dedicated computers and cannot be taken away from the lab, so unfortunately they cannot be made available. The zip archive contains an example used in the interim report on which the subsequent digital twin models were developed. The zip archive includes the dynamics simulation model of the Stewart platform, the code for calculating the inverse solution and the Jacobian matrix, the set of platform kinematics parameters, and the model of the Stewart platform in Solidworks. Sorry again.
