# Supplementary figures and images for: Modeling and implementation of a real-time digital twin for the Stewart platform with real-time trajectory computation
Source: PeerJ Comput Sci. 2025 May 20;11:e2892. doi: 10.7717/peerj-cs.2892 (PMC12192767; doi:10.7717/peerj-cs.2892)

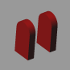

Supplement: Supplemental Information 1 [file peerj-cs-11-2892-s001.zip › StewartPro Shadow/StewartPlatformSupport/Images/Coupling.png]

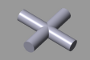

Supplement: Supplemental Information 1 [file peerj-cs-11-2892-s001.zip › StewartPro Shadow/StewartPlatformSupport/Images/Crosspiece.png]

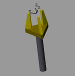

Supplement: Supplemental Information 1 [file peerj-cs-11-2892-s001.zip › StewartPro Shadow/StewartPlatformSupport/Images/EndEffector.png]

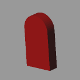

Supplement: Supplemental Information 1 [file peerj-cs-11-2892-s001.zip › StewartPro Shadow/StewartPlatformSupport/Images/Flange.png]

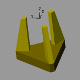

Supplement: Supplemental Information 1 [file peerj-cs-11-2892-s001.zip › StewartPro Shadow/StewartPlatformSupport/Images/Gripper.png]

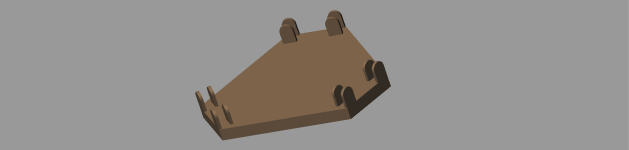

Supplement: Supplemental Information 1 [file peerj-cs-11-2892-s001.zip › StewartPro Shadow/StewartPlatformSupport/Images/HexagonPlate.png]

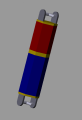

Supplement: Supplemental Information 1 [file peerj-cs-11-2892-s001.zip › StewartPro Shadow/StewartPlatformSupport/Images/Leg.png]

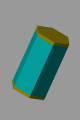

Supplement: Supplemental Information 1 [file peerj-cs-11-2892-s001.zip › StewartPro Shadow/StewartPlatformSupport/Images/LegShaft.png]

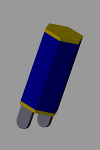

Supplement: Supplemental Information 1 [file peerj-cs-11-2892-s001.zip › StewartPro Shadow/StewartPlatformSupport/Images/LowerLeg.png]

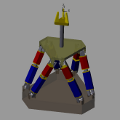

Supplement: Supplemental Information 1 [file peerj-cs-11-2892-s001.zip › StewartPro Shadow/StewartPlatformSupport/Images/Manipulator.png]

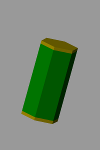

Supplement: Supplemental Information 1 [file peerj-cs-11-2892-s001.zip › StewartPro Shadow/StewartPlatformSupport/Images/MiddleLeg.png]

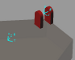

Supplement: Supplemental Information 1 [file peerj-cs-11-2892-s001.zip › StewartPro Shadow/StewartPlatformSupport/Images/PlateCoupling.png]

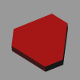

Supplement: Supplemental Information 1 [file peerj-cs-11-2892-s001.zip › StewartPro Shadow/StewartPlatformSupport/Images/SkewedHexagon.png]

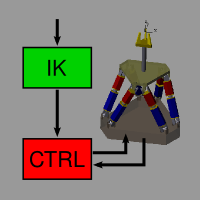

Supplement: Supplemental Information 1 [file peerj-cs-11-2892-s001.zip › StewartPro Shadow/StewartPlatformSupport/Images/System.png]

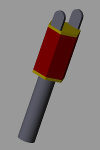

Supplement: Supplemental Information 1 [file peerj-cs-11-2892-s001.zip › StewartPro Shadow/StewartPlatformSupport/Images/Upperleg.png]
